# Perspective of Undergraduate Dental Students on Horizontal and Vertical Integrations in Prosthodontics Education: A Mixed‐Methods Approach

**DOI:** 10.1155/ijod/9095160

**Published:** 2026-08-12

**Authors:** Watcharapong Mongkolrattanasit, Veerit Tanvarasethee, Supachai Chuenjitwongsa, Nareudee Limpuangthip

**Affiliations:** ^1^ Department of Oral Pathology, Faculty of Dentistry, Chulalongkorn University, Bangkok, Thailand, chula.ac.th; ^2^ Department of Prosthodontics, Faculty of Dentistry, Chulalongkorn University, Bangkok, Thailand, chula.ac.th; ^3^ Department of Biochemistry, Faculty of Dentistry, Chulalongkorn University, Bangkok, Thailand, chula.ac.th; ^4^ Dental Education Unit, Faculty of Dentistry, Chulalongkorn University, Bangkok, Thailand, chula.ac.th

**Keywords:** curriculum integration, dental education, knowledge, learning, prosthodontics

## Abstract

**Objectives:**

Curricular integration in dental education is essential for bridging basic sciences into clinical practices. This study aimed to evaluate dental students’ perceptions of the current prosthodontic curriculum structure in a Thai undergraduate dental program, regarding horizontal and vertical integration, by applying a mixed‐methods approach to examine possible connections among courses.

**Materials and Methods:**

A mixed‐methods approach was employed. A quantitative questionnaire was distributed to fifth‐ and sixth‐year undergraduate dental students and recent graduates (*n* = 286), asking them to rate the importance of subjects related to prosthodontics education on a four‐level ordinal scale (from nonessential to essential [score 0–3]). Friedman’s two‐way analysis of variance by ranks was employed, and the differences in mean rank values were utilized to develop an integration framework of subjects. Additionally, focus group interviews were conducted with nine clinical students and seven recent graduates to explore their perspectives on subject integration in detail.

**Results:**

The response rate of the online questionnaire was 72.0%, and 16 dental students and recent graduates participated in a focus group interview. The horizontal and vertical integration structure for dental prosthodontics education in undergraduate programs was proposed, with highly essential subjects for vertical integration, including occlusion, dental materials, oral examination, oral anatomy, and general medicine. Moderately essential subjects were included in both vertical and horizontal integrations, particularly other clinical courses.

**Conclusions:**

Based on the students’ perspectives at a single institution, prosthodontic education appeared to demonstrate stronger vertical integration across different stages of the undergraduate dental curriculum than horizontal integration across disciplines taught within the same stage. Students perceived fewer connections between prosthodontics and other disciplines, suggesting opportunities to strengthen interdisciplinary integration.

## 1. Introduction

The interdisciplinary integration in dental education is fundamental to modern curriculum design, ensuring a seamless transition between basic sciences and clinical practice [1, 2]. Formerly, the isolated subject compartments led to fragmented knowledge, requiring students to synthesize disparate concepts on their own [3]. Currently, undergraduate dental programs have shifted from rigid, discipline‐based structures toward competency‐based learning [4], favoring curricular integration to cultivate critical thinking, problem‐solving, and clinical competency [5]. Curricular integration in dental education includes horizontal and vertical integration, bridging the gap between basic sciences and clinical practice while enhancing student engagement and patient‐centered care [6]. Vertical integration refers to the connection and reinforcement of knowledge, skills, and competencies across different years or stages of training, including links between foundational and clinical learning. This integration facilitates the progression of knowledge from basic sciences to clinical practice through early and continuous clinical exposure, enabling students to apply theoretical concepts in real clinical settings [2, 7, 8]. Meanwhile, horizontal integration refers to the coordination and linkage of disciplines taught concurrently within the same stage of the curriculum, promoting connections among subjects with related themes or learning objectives [2, 8, 9].

In Thailand, undergraduate dental education is a 6‐year curriculum, including 3 years of preclinical training, followed by 3 years of clinical practice [10]. Dental prosthodontics for the undergraduate curriculum is divided into fixed and removable prosthodontics, which involve training in the fabrication of removable partial and complete dentures. Prosthodontic lectures and laboratory practice extend from the third to the fourth year, starting from the principles of complete denture fabrication, followed by concurrent education in removable partial dentures and fixed prosthodontics. Prosthodontic clinical practice takes place during the fifth and sixth years. Competent dentists should possess the necessary knowledge, skills, and attitude for patient treatment. However, students often experience difficulties in learning and practicing to achieve the appropriate prosthodontic competency. Given the complexity of prosthodontic treatment, students often encounter challenges in achieving the competencies required for patient care [4, 10, 11]. Therefore, an integrated curriculum design should enable dental students to develop clinical competence in these fields.

Recent advances in dental education, including digital dentistry, augmented reality, virtual reality, and artificial intelligence, have transformed how students acquire and apply knowledge and skills [12, 13]. The innovations require learners to integrate concepts across disciplines and apply foundational knowledge in increasingly complex clinical contexts, highlighting the importance of both horizontal and vertical curriculum integration [14]. Despite the growing adoption of integrated curricula, several dental programs continue to separate basic sciences from clinical training and rely on discipline‐based teaching approaches, limiting meaningful connections between theoretical instruction and clinical practice [5, 6, 15]. The lack of clear linkages among courses that demonstrate horizontal and vertical integration in dental education remains a significant barrier, requiring further research to optimize curricular design [1]. Despite the growing adoption of integrated curricula, many dental programs continue to separate basic sciences from clinical training and rely on discipline‐based teaching approaches, limiting meaningful connections between theoretical instruction and clinical practice [4, 5, 12]. The lack of clearly defined horizontal and vertical connections among courses remains a challenge in dental education and warrants further investigation to inform curriculum development.

Previous studies have addressed horizontal and vertical integration in dental schools [2], focusing on dental materials [4], conservative oral healthcare [16], and comprehensive patient care courses [17]. However, to our knowledge, no relevant studies have focused on dental prosthodontics combined with substantial amounts of content requiring mapping of clinical and basic sciences through a structural sequence. To address this challenge, the aim of this study was to evaluate dental students’ perceptions of the current prosthodontic curriculum structure in a Thai undergraduate dental program, regarding horizontal and vertical integration, by applying a mixed‐methods approach to examine the possible connections among courses.

## 2. Materials and Methods

### 2.1. Participants and Study Design

This study adopted a critical theory paradigm and explanatory mixed‐method design, employing a quantitative method to gather data trends and a qualitative method to explain the quantitative findings. The eligible participants involved fifth‐ and sixth‐year dental students from the undergraduate program at the Faculty of Dentistry, Chulalongkorn University, during the 2022 academic year and recent dental graduates. The participants comprised clinical students with clinical experience in prosthodontic practice as well as recent graduates who completed their studies within the past 2 years. Participants who declined to complete the questionnaire were excluded from the study.

The study was conducted from December 2022 to April 2023 during the prosthodontic courses and consisted of three components: documentary analysis, quantitative online questionnaires, and qualitative focus group interviews. Ethical approval was obtained from the Human Research Ethics Committee of the Faculty of Dentistry (HREC‐DCU 2022‐115).

### 2.2. Quantitative Data Collection

Using the dental school’s curriculum themes (File S1) along with prosthodontic course structures and learning topics (File S2) as a framework, a documentary analysis of course syllabi across all 6 years of the undergraduate dental program and a review of the literature on curriculum integration were undertaken. The findings informed the development of an online questionnaire (File S3). The questionnaire statements were subsequently content‐validated by two prosthodontic faculty members through expert review and item revision, followed by a pilot study involving 13 clinical students to ensure clarity and relevance. The questionnaire included demographic information and key questions designed to identify the subjects necessary for understanding and achieving success in patient treatment with fixed and removable prosthodontics. Participants rated subjects on a four‐level ordinal scale: highly essential (score = 3), moderately essential (score = 2), less essential (score = 1), and nonessential (score = 0).

Data from the questionnaire were analyzed using a statistical software package (SPSS version 29.0). Descriptive statistics provided an overview of the sample data. To assess the perceived essentiality of the surveyed subjects, a nonparametric Friedman’s two‐way analysis of variance by ranks was employed, followed by Bonferroni‐adjusted pairwise comparisons. Based on the results of these comparisons, subjects were grouped into hierarchical tiers according to the similarity of their mean ranks. Subjects within the same tier did not differ significantly from one another, whereas subjects belonging to different tiers exhibited significant differences. The resulting tiers were interpreted as representing highly essential, moderately essential, and less essential subjects, with higher mean ranks indicating greater perceived essentiality.

### 2.3. Qualitative Data Collection

The qualitative phase was conducted to explain and validate the quantitative findings by exploring students’ perceptions of vertical and horizontal integration among subjects and by providing deeper insights into the patterns identified from the ranking data. The qualitative data were analyzed using framework analysis, whereby themes and relationships among subjects were organized and interpreted according to established concepts of curriculum integration and relevant educational theories to generate a semi‐structured, open‐ended questionnaire for focus group interviews (File S4).

Focus group interviews were conducted with nine clinical dental students and seven recent graduates to explore the perceptions of subject integration within the curriculum. Participants were recruited through purposive sampling from volunteers who consented after the study objectives were explained. Interviews were conducted by two investigators (Watcharapong Mongkolrattanasit and Veerit Tanvarasethee), who were trained by a qualitative research expert (Supachai Chuenjitwongsa). All interviews were conducted in Thai, and the findings were translated into English. The translations were cross‐validated by two investigators (Watcharapong Mongkolrattanasit and Veerit Tanvarasethee), with additional verification by a prosthodontist (Nareudee Limpuangthip), a dental education specialist (Supachai Chuenjitwongsa), and two native English‐speaking experts to ensure accuracy and consistency. Data collection continued until thematic saturation was achieved.

Transcribed data were coded and analyzed using framework analysis to classify subjects into vertical and horizontal integration in prosthodontic education. Curriculum integration was categorized according to established conceptual definitions rather than predefined quantitative thresholds. Horizontal integration was defined as the coordination and linkage of disciplines taught concurrently within the same stage of the curriculum, whereas vertical integration was defined as the connection and reinforcement of knowledge, skills, and competencies across different years or stages of training, including the integration of foundational and clinical learning [2, 8]. Threshold concepts, tacit knowledge, and other educational theories were used as the frameworks to categorize vertical and horizontal relationships. Threshold concepts were characterized by features such as being transformative, integrative, irreversible, troublesome, bounded, discursive, and involving a liminal stage of learning. In particular, the integrative characteristic enabled learners to recognize previously hidden relationships among concepts and disciplines. Coding and framework development were conducted independently by two investigators (Watcharapong Mongkolrattanasit and Veerit Tanvarasethee) and subsequently verified by a prosthodontist (Nareudee Limpuangthip) and a dental education specialist (Supachai Chuenjitwongsa).

## 3. Results

A response rate of 72.0% (206 responses) was obtained from 286 eligible participants who were distributed the online questionnaire. Response rates varied across cohorts, with the highest participation observed among sixth‐year dental students (85.2%, 75/88), followed by recent graduates pursuing higher education (81.3%, 26/32), fifth‐year dental students (70.7%, 58/82), and recent graduates (56.0%, 47/84). Table 1 presents the demographic characteristics of the respondents, whose mean age was 25.1 years (SD = 1.9). Based on the provided mean ranks and pairwise comparison test (Table 2), the subjects can be categorized into three groups reflecting their perceived essentiality or strength of subject connection for success in prosthodontic treatment: highly essential (strong connection), moderately essential (moderate connection), and less essential (weak connection). Subjects within each group did not have statistically significant differences in their mean ranks. Although minor differences in mean rank values were observed between clinical students and recent graduates, the classification of subjects into distinct levels of perceived connections remained consistent across groups.

**Table 1:** Characteristics of the respondents (*n* = 206).

Variables	Clinical students (*n* = 133): *n* (%)	Graduates (*n* = 73): *n* (%)
Sex		
Male	59 (44.4)	31 (42.5)
Female	74 (55.6)	42 (57.5)
Educational level		
Fifth‐year students	58 (43.8)	—
Sixth‐year students	75 (56.2)	—
Recent graduates	—	47 (64.4)
Recent graduates pursuing higher education	—	26 (35.6)

**Table 2:** Levels of connection between prosthodontics and other subjects based on mean ranks and pairwise comparison test.

Subjects	Levels of connection	Mean rank values		
		Overall	Clinical students	Recent graduates
Occlusion Dental materials Oral examination Oral anatomy General medicine	Strong	13.6 12.4 12.2 12.0 11.6	13.6 12.5 12.0 11.8 11.5	13.8 12.8 13.2 12.7 11.6

Radiology Oral surgery Dental anatomy Restorative dentistry Periodontology Endodontics Orthodontics Oral medicine	Moderate	9.0 9.0 8.6 8.5 8.1 7.6 7.5 7.4	9.2 9.1 8.3 8.6 7.8 7.5 7.2 7.4	9.7 8.6 8.8 8.1 9.1 7.8 7.1 7.6

Basic histology Oral pathology Community dentistry Epidemiology	Weak	5.9 5.8 4.1 3.0	6.2 6.1 3.7 3.1	4.5 4.5 4.6 2.2

Physiology, biochemistry		>80% reported not essential at all		

*Note*: No statistically significant difference between subjects within the same level (*p* > 0.05). Pediatric dentistry was excluded, as the specialty encompasses a combination of multiple clinical disciplines.

The quantitative findings were further examined through qualitative inquiry to confirm and contextualize the perceived connections among the subjects. The framework analysis of focus group interviews with clinical students and recent graduates identified both vertical and horizontal relationships among prosthodontics‐related subjects, which were subsequently categorized into three hierarchical levels:

1. Basic sciences: covering physics, biochemistry (biology, molecular biology, and chemistry), histology, human anatomy, oral anatomy, and physiology.
2. Preclinical or basic dental sciences: including pharmacology, general medicine, oral pathology, radiology, oral examination, occlusion, dental materials, community dentistry, and epidemiology.
3. Clinical dental sciences: encompassing endodontics, restorative dentistry, orthodontics, oral surgery, oral medicine, pediatric dentistry, periodontics, and prosthodontics.

**Figure 1:**
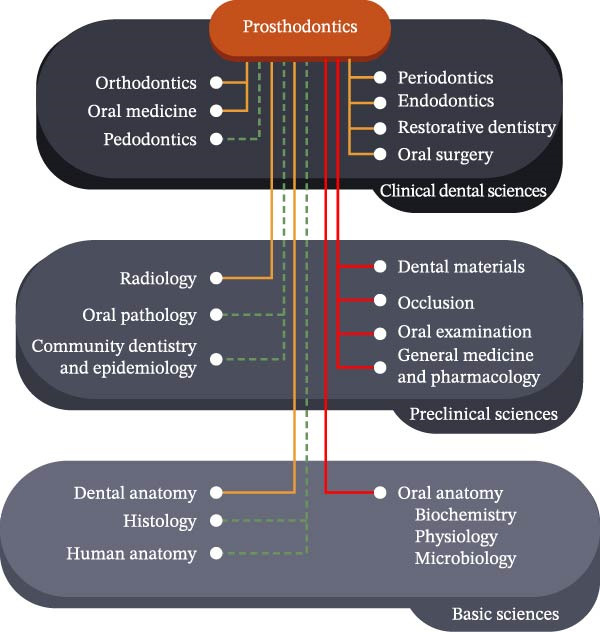
Vertical and horizontal integration between subjects. Red lines indicate highly essential (strong connections); orange lines indicate moderately essential (moderate connections); green dashed lines indicate less essential (weak connections); subjects without connecting lines indicate that 80% of participants reported them as not essential at all.

Figure 1 illustrates the vertical and horizontal integration of curriculum structures related to prosthodontics, as well as the strength of connections between subjects in undergraduate dental education. Vertical integration indicates knowledge progression from basic sciences to clinical practice, while horizontal integration represents the connections between subjects within the same academic level. The analysis identified key themes related to the relevance of foundational knowledge, clinical applications, and interconnectivity between disciplines. It was noted that pediatric dentistry was excluded as the specialty encompasses a combination of multiple clinical disciplines.

### 3.1. Vertical Integration

#### 3.1.1. Basic Sciences

The participants highlighted the importance of basic sciences, particularly oral and dental anatomy, in understanding prosthodontic principles. Oral anatomy was recognized as crucial for denture fabrication, influencing force distribution and impression techniques. Students stated that understanding oral structures assists in assessing border molding boundaries, which is fundamental to achieving optimal denture retention:

*“Understanding oral anatomy helps identify areas where forces can be effectively applied and tolerated. This is also crucial for selecting impression-making techniques.”* (fifth‐year student)

Similarly, dental anatomy and occlusion were seen as essential for designing occlusion and articulation, particularly in crown fabrication and tooth arrangement:

*“Learning dental anatomy helps me understanding the ideal tooth form. Laboratory practicing allows me to visualize its actual appearance while concurrently developing my hand skill. This supports continuous learning in both restorative dentistry and fixed dental prosthodontics.”* (sixth‐year student)

#### 3.1.2. Preclinical Sciences

The significance of preclinical subjects that influence prosthodontic practice included dental materials, occlusion, oral examination, and general medicine. However, there was divergence in opinion regarding the timing of these subjects being taught in the curriculum. Some students expressed difficulty appreciating dental materials theory before having clinical exposure, suggesting that learning about dental materials applications too early may lead to forgotten and unretrievable knowledge. A deeper understanding was achieved after clinical exposure as students could better relate theoretical knowledge to real‐world applications:

*“When studying prosthodontics, I often refer back to dental materials. However, I believe it is too early to learn about dental materials before gaining real clinical experience.”* (fifth‐year student)

*“Revisiting dental materials course in the final year after clinical exposure allows a deeper understanding and more comprehensive summary based on prior clinical experience.”* (sixth‐year student)

The importance of occlusion in prosthodontic treatment planning was that concepts of dental occlusion should precede prosthodontic courses:

*“Studying occlusion terminology, such as centric relation and occlusion schemes, beforehand significantly improves our comprehension of prosthodontic concepts.”* (fifth‐year student)

*“Occlusion affects treatment in various aspects; it would be beneficial to study occlusion before prosthodontics.”* (fifth‐year student)

*“Occlusion impacts prosthodontics at every stage, from treatment planning to the selection of equipment and prosthetic design.”* (sixth‐year student)

Students acknowledged that a comprehensive oral examination of abutment teeth in prosthodontics requires comprehensive knowledge from both periodontics and endodontics. Failure to identify periodontal or endodontic complications before prosthetic rehabilitation could lead to treatment failure:

*“Inadequate examination can lead to missing critical issues, such as failing to identify teeth that require endodontic treatment, which can compromise prosthetic success.”* (recent graduate)

*“Understanding periodontal health is crucial for planning prosthodontic treatment and predicting the longevity of prostheses.”* (sixth‐year student)

In addition to oral and dental‐related knowledge, general medicine is essential, particularly for evaluating patients’ systemic conditions, to ensure comprehensive treatment planning and consideration of overall health.

*“Patient evaluation, including assessment of underlying conditions, readiness for treatment, and case selection, is crucial in prosthodontics, as treatment planning must consider the patient holistically, not just their oral condition. Additionally, soft skills and communication with patients are also important.”* (recent graduate)

*“Systematic patient evaluation is essential, as it directly influences prosthodontics treatment planning.”* (recent graduate)

*“Learning general medicine enables safe patient care by helping avoid procedures that could be harmful the patient’s overall condition.”* (sixth‐year student)

### 3.2. Horizontal Integration

#### 3.2.1. Horizontal Connections With Other Clinical Sciences Subjects

During the progress to clinical training, the students recognized the interconnections between prosthodontics and other clinical science disciplines. For example, participants emphasized that restorative dentistry provides essential hand skills that facilitate crown preparation, and endodontic knowledge and practice were also seen as integral to post‐core restorations:

*“If you practice your hand skills and understand tooth preparation in restorative dentistry well, it will make preparing crowns much easier.”* (recent graduate)

*“If I had performed post and core procedures in my fifth year without any experience in endodontic treatment on real patients, I would have drilled with less confidence.”* (recent graduate)

Some students noted that preprosthetic oral surgery procedures, such as vestibuloplasty and bone preparation, as well as periodontics, significantly influence treatment planning for removable prostheses:

*“Preprosthetic surgery knowledge is essential for denture treatment, including the healing of extracted sites and bone preparation for future prosthetic work.”* (fifth‐year student)

*“Patients who choose removable dentures often have specific oral conditions, such as significant tooth mobility, that require extractions. Understanding periodontal health is crucial for treatment planning and estimating longevity of the prostheses.”* (sixth‐year student)

#### 3.2.2. Horizontal Connections Within Prosthodontic Subjects

Students expressed that learning prosthodontic subjects in parallel facilitates better knowledge retention. Aligned teaching between different prosthodontic sub‐disciplines, particularly fixed and removable prosthodontics, is essential:

*“Studying and working on the complete denture and removable partial denture labs concurrently allows more rapid understanding and completion of tasks.”* (fifrth‐year student)

*“There is a significant linkage between prosthodontic subjects, such as surveyed crowns and single dentures, which require integration of fixed and removable prosthodontics.”* (recent graduate)

Moreover, students found that managing various cases simultaneously in clinical practice forced them to reflect on the treatment rationale and decision‐making, emphasizing the importance of integrated learning:

*“Managing several cases simultaneously requires self-reflection on why different methods are used for similar cases. Understanding the rationale behind selecting specific approaches is essential for effective treatment planning.”* (sixth‐year student)

## 4. Discussion

Participants from different stages of training were included in this study to capture diverse perspectives on curriculum integration. The quantitative findings were used to identify overall patterns, whereas the qualitative phase was conducted to explore in depth and confirm the perceived vertical and horizontal relationships among the subjects. The present findings highlight the significance of connections between subjects related to prosthodontics in the undergraduate dental curriculum. By integrating basic, preclinical, and clinical sciences, both vertical and horizontal integrations create a progressive and cohesive learning experience, allowing students to adopt theoretical knowledge and skills into professional competencies necessary for prosthodontic practice. The findings underscore the importance of vertical integration as a fundamental to advancing learning and practice, creating a strong connection. Meanwhile, horizontal integration facilitates easier learning across various subjects, resulting in moderate‐to‐weak connections.

Vertical and horizontal integration are key strategies for improving learning efficiency and clinical preparedness in dental education [4]. From the present findings, vertical integration has shown strong connections with prosthodontics, whereas horizontal integration often demonstrates moderate‐to‐weak relationships. Vertical integration aligns with constructivist learning theories, which propose that students actively construct knowledge through experience and contextual engagement [18]. For example, oral examination subjects should integrate comprehensive principles, such as endodontic and periodontal assessments, to support prosthodontic evaluation and treatment planning.

Vertical integration offers significant benefits in addressing fragmented learning challenges in dental education, particularly the issue of scatter learning, where courses are disconnected and taught prematurely. These result in surface learning and subsequent forgetting. Without reiteration, clinical contextualization, or practical application, newly introduced information or skills, especially surface‐level learning, are rapidly forgotten [19]. This is in contrast to threshold concepts that once understood are deeply retained as they involve deep‐level learning [20]. Vertical integration mitigates these issues by demonstrating interdisciplinary interconnection and ensuring a logical content sequence. For example, introducing dental materials too early prior to clinical exposure increases the risk of surface learning and subsequent forgetting, whereas revisiting these concepts through advanced courses such as complete denture laboratories and fixed prosthodontics, studied in the following years, reinforces foundational knowledge within clinically relevant frameworks. This approach effectively bridges basic sciences and clinical practice, transforming theoretical concepts into professional competencies.

In contrast, horizontal integration refers to the connection between disciplines within the same academic year, encouraging cross‐disciplinary collaboration to improve comprehensive treatment planning and clinical decision‐making. This is often reflected in the dental curriculum design, where laboratory sessions and clinical practices are strategically scheduled within the same academic year. In some cases, similar topics from different subjects are deliberately arranged to occur on the same day or within the same week. This instructional strategy is referred to as temporal coordination [21]. This study highlights the importance of horizontal integration as subjects at the same level are already positioned within the same academic year without identifying significant issues. However, in prosthodontics, horizontal integration should focus on linking subdisciplines, such as removable and fixed prosthodontics, to enhance learning coherence and clinical application.

To enhance prosthodontic education, curriculum revision should focus on strengthening vertical and horizontal integration. Vertical integration can be improved by implementing educational strategies that support students applying theoretical knowledge to clinical scenarios from the early years of their curriculum [2, 5]. Some students have reported difficulty retaining preclinical knowledge into their clinical years, suggesting that traditional didactic instruction lacks relevance when not immediately contextualized in patient care [5]. The strategies should provide effective scaffolding techniques that bridge theoretical knowledge with practical applications [22]. The constructivist approach supports early clinical exposure and interdisciplinary case discussions, reinforcing that students learn best when applying theoretical knowledge in practical situations [23]. This perspective is particularly relevant in prosthodontic education, where hands‐on learning fosters deeper comprehension of complex procedures.

Early clinical exposure is another essential strategy to enhance integration, where students actively participate in prosthodontic procedures alongside experienced faculty mentors, reinforcing both cognitive and procedural competencies [24]. This approach is supported by situated learning theory, which suggests that students learn more effectively when knowledge is embedded in authentic clinical settings [25]. Early exposure to clinical applications through vertical integration enables students to apply basic science concepts in real‐world clinical contexts [7].

Meanwhile, horizontal integration across related disciplines strengthens conceptual understanding and interdisciplinary decision‐making [15]. For instance, during a preclinical dental materials course, students assist in prosthodontic clinics by mixing impression materials for the senior clinical students. This strategy facilitates the retrieval and application of dental material knowledge in authentic clinical contexts, consistent with the cognitivist approach, while simultaneously fostering learning through dialogue and collaboration with senior peers, aligning with the constructivist approach [18].

Apart from curriculum structures, it is the instructors’ responsibility to consult each other and harmonize between different subjects and different parts of the same subject. Preclinic instructors should connect their teachings to clinical applications, while clinical instructors should refer back to preclinical knowledge during case discussions to demonstrate interconnections and facilitate the proper adoption of prior knowledge. To support this, dental schools should establish a core team responsible for course arrangement to ensure an optimal structural sequence and avoid repetitive content [21, 26].

This study was the first to provide valuable insights into vertical and horizontal integration between subjects related to prosthodontics in the dental curriculum. The study protocol approach could serve as a useful guide for other institutions seeking to examine, develop, and refine curriculum integration within their own educational contexts. The exclusion of other subjects does not imply their insignificance but rather reflects their indirect relevance to prosthodontics, aligning them more closely with other dental fields.

Some limitations are noted in this study. The findings are based on data collected at a single institution, which may limit their generalizability to educational settings in other countries with differing curricula. Nevertheless, the aim of this explanatory mixed‐methods study was to provide an in‐depth exploration of students’ perceptions of curriculum integration within a specific context rather than to generate findings intended for broad generalization. This study evaluated curriculum integration from the perspectives of students and recent graduates. However, curriculum integration is a multifaceted construct that may be perceived differently by other stakeholders, including faculty members, curriculum developers, educators, and practicing dentists. In addition, the study assessed perceived curriculum integration rather than educational outcomes; therefore, its impact on knowledge retention, clinical performance, and competence needs further exploration. The cross‐sectional study design captures students’ perspectives at a single point in time without assessing the long‐term impact of integration on clinical competence. Although comparisons with international dental curricula may provide broader perspectives, meaningful comparisons are limited by differences in educational, regulatory, and institutional contexts. Therefore, the findings should be interpreted within the context of the studied institution. Future multicenter and cross‐national studies incorporating diverse stakeholder perspectives and complementary research methodologies are warranted to provide more comprehensive evaluations and comparisons of curriculum integration. Future research should employ longitudinal designs to evaluate how curricular integration influences student learning and professional competence over time while incorporating perspectives from both students and professional providers.

## 5. Conclusion

Based on the students’ perspectives at a single institution, prosthodontic education appeared to demonstrate stronger vertical integration across different stages of the undergraduate dental curriculum than horizontal integration across disciplines taught within the same stage. Students perceived fewer connections between prosthodontics and other disciplines, suggesting opportunities for strengthening interdisciplinary integration.

## Author Contributions

Watcharapong Mongkolrattanasit conceptualized the idea, collected and analyzed data, interpreted the findings, wrote the original draft, and edited the manuscript. Veerit Tanvarasethee conceptualized the idea, collected and analyzed data, interpreted the findings, prepared illustrated works, and edited the manuscript. Supachai Chuenjitwongsa and Nareudee Limpuangthip conceptualized the idea and study design, validated data, interpreted the findings, wrote the original draft, and edited the manuscript.

## Funding

The study was supported by Faculty Research (Grant DRF69_020), Faculty of Dentistry at Chulalongkorn University, Bangkok, Thailand.

## Disclosure

All authors reviewed the final manuscript, read and approved the final version of the manuscript for submission, and agreed to be accountable for all aspects of the work. The corresponding authors have full access to all of the data in this study and take complete responsibility for the integrity of the data and the accuracy of the data analysis.

## Ethics Statement

The study had been performed in accordance with the Declaration of Helsinki and was approved by the Human Research Ethics Committee of the Faculty of Dentistry, Chulalongkorn University (Code Number HREC‐DCU 2022‐115). All participants agreed to participate and signed informed consent prior to study participation.

## Conflicts of Interest

The authors declare no conflicts of interest.

## Supporting Information

Additional supporting information can be found online in the Supporting Information section.

## Supporting information


**Supporting Information 1** File S1: Curriculum themes of the Doctor of Dental Surgery (DDS) program at the Faculty of Dentistry, Chulalongkorn University.

**Supporting Information 2** File S2: Prosthodontic course structures and learning topics, and other courses in the Thai undergraduate curriculum.

**Supporting Information 3** File S3: Survey questionnaires.

**Supporting Information 4** File S4: Focus group interview questions.

## Data Availability

The dataset generated during the current study is available upon request to the corresponding authors.
