# Supplementary material for: Perspective of Undergraduate Dental Students on Horizontal and Vertical Integrations in Prosthodontics Education: A Mixed‐Methods Approach
Source: Int J Dent. 2026 Aug 12;2026:9095160. doi: 10.1155/ijod/9095160 (PMC13469755; doi:10.1155/ijod/9095160)

Curriculum Themes of the DDS Programme, Faculty of Dentistry, Chulalongkorn University, Thailand

Originally developed in 2004 by **Professor Thanapoom Osathanon**, Originally implemented in 2007 by **Assistant Professor Janejira Thirawat**, Revised in 2020 by **Associate Professor Sirivimol Srisawasdi** and **Assistant Professor Supachai Chuenjitwongsa**

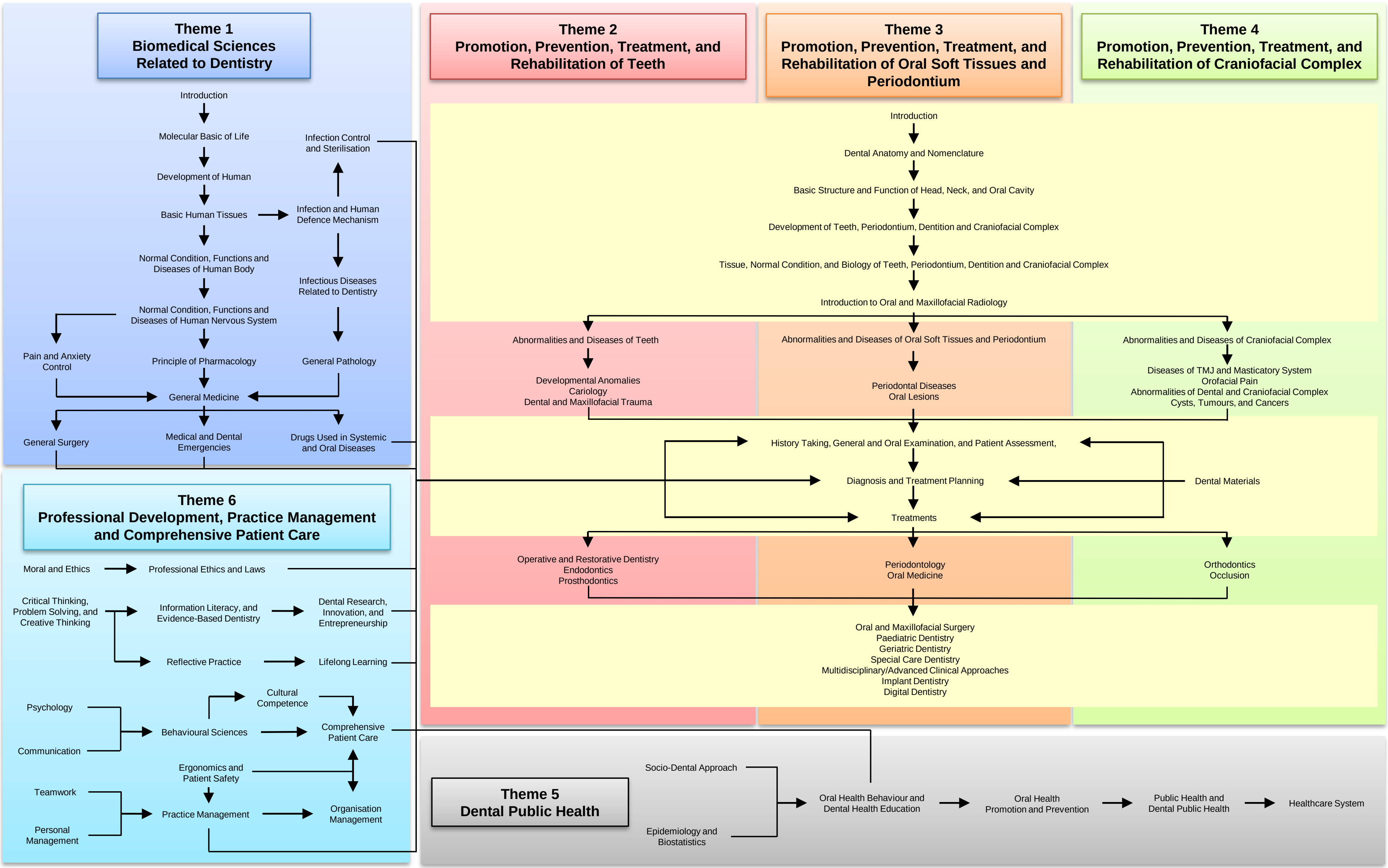

Supplement: Supplementary file 1 [file IJOD-2026-9095160-s001.pdf]
