# Supplementary material for: Perspective of Undergraduate Dental Students on Horizontal and Vertical Integrations in Prosthodontics Education: A Mixed‐Methods Approach
Source: Int J Dent. 2026 Aug 12;2026:9095160. doi: 10.1155/ijod/9095160 (PMC13469755; doi:10.1155/ijod/9095160)
Supplement: Supplementary file 2 [file IJOD-2026-9095160-s002.pdf]

Supplementary File 2. Prosthodontic course structures and learning topics, and other courses in Thai undergraduate curriculum

Year (semester)	Prosthodontic courses						Other courses
	Complete Denture		Removable Partial Denture		Fixed prosthodontics		
	Lecture	Laboratory	Lecture	Laboratory	Lecture	Laboratory	
**1 (1)**	None		None		None		Basic sciences
**1 (2)**	None		None		None		Biomedical sciences (Cell biology, Molecular biology)
**2 (1)**	None		None		None		- Dental anatomy<br>- Head neck anatomy<br>- Histology<br>- Physiology
**2 (2)**	None		None		None		- Radiology<br>- Restorative dentistry
**3 (1)**	● Introduction<br>- Overview & Definition<br>● Basic theories<br>- Oral anatomy<br>- Biomechanics<br>- Impression theory & techniques<br>- Posterior palatal seal<br>- Maxillomandibular relation<br>- Articulator	**Lab I:**<br>- Custom tray<br>- Baseplate and occlusion rim<br>- Mounting in articulator<br>- Anterior teeth arrangement	None		None		- Pathology<br>- Restorative (Lab)<br>- Periodontology
**3 (2)**	● Basic theories (continued)<br>- Denture esthetics<br>- Tooth selection<br>- Occlusal scheme<br>● Denture fabrication<br>- Denture processing<br>- Lab remount and selective grinding	**Lab II**:<br>- Posterior teeth arrangement & waxing<br>- Denture processing<br>- Lab remount and selective grinding	● Introduction<br>- Overview & Classification<br>2. Basic theories<br>- RPD components<br>- Acrylic-based partial denture<br>- Flexible denture	**Lab I:**<br>- Surveying<br>- Bite registration and mounting<br>- Wire clasp bending<br>- Teeth arrangement<br>- Denture processing	● Introduction<br>● Basic theories<br>- Principles of tooth preparation, finishing line<br>- Dental material for fixed partial dentures<br>- Impression making<br>- Pontic & substructure design<br>● Laboratory procedures:<br>- Die and working model<br>- Spruing, Investing, Casting	**Lab I:**<br>- Tooth preparation for full metal crown<br>- Tooth preparation for porcelain-fused to metal crown<br>- Final impression<br>- Die and working model<br>- Wax pattern	- Occlusion<br>- Oral examination<br>- Endodontology<br>- Oral surgery<br>- Restorative lab<br>- Periodontic lab
**4 (1)**	None		● Basic theories (continued)<br>- RPD principles & biomechanics<br>- Survey, path of insertion, guiding planes	**Lab II:**<br>- repair<br>- design<br>- direct retainer preparation	● Basic theories on post/core/ crown<br>- Principles of pin-tooth<br>- Cast post and core<br>- Conventional cement	**Lab II:**<br>- sprueing investing casting<br>- tooth preparation for all ceramic	- Restorative (clinic)<br>- endodontics (lab)<br>- periodontics (clinic)<br>- oral surg (clinic)<br>- oral examination (clinic)

			- Denture movements<br>- Impression techniques	- tooth alteration<br>- wax pattern<br>- alginate impression	- Resin cement<br>- Prefabricated post<br>● Laboratory and clinical procedures:<br>- Provisional restoration and temporary cement<br>- Clinical evaluation and adjustment<br>- Soldering<br>- Porcelain application	- try-in restorations<br>- soldering<br>- temporary crown<br>- cast post, prefabricated post & core<br>- canal impression<br>- waxing cast pose	- radiology (clinic)
**4 (2)**	● Clinical applications<br>- Oral exam<br>- Impression<br>- Occlusion rim<br>- Recording maxilla-mandibular relation<br>- Occlusion<br>- Denture delivery, Post insertion problems<br>● Advanced topics<br>- Immediate complete denture<br>- Overdenture<br>- Single denture<br>- Metal base complete denture<br>- Implant retained/supported denture	None	● Denture design<br>- Abutment selection<br>- Direct & indirect retainers<br>- Framework<br>● Clinical wrap-up<br>- Oral examination<br>- Pre-prosthetic treatment<br>- Survey and design<br>- Tooth preparation<br>- Impression<br>- Framework fitting<br>- Functional impression<br>- Occlusion<br>- Delivery<br>- Rebase Reline Repair<br>● Advanced topics<br>- Removable bridge<br>- Interim partial denture<br>- Maxillofacial prosthesis	None	● Clinical applications<br>- Oral examination and tx plan<br>- Preparation<br>- Occlusion<br>- Survey crown<br>- Lab communication<br>- Clinical failure<br>● Advanced topics<br>- Cantilever bridge<br>- Digital workflow<br>- Smile analysis<br>- Implant<br>- Inlay / Onlay	None	- restorative (clinic)<br>- endodontics (clinic)<br>- periodontics (clinic)<br>- oral surgery (clinic)<br>- oral examination (clinic)
**5 (1 & 2)**	**---------- Prosthodontic clinic ----------**						- restorative (clinic)<br>- endodontics (clinic)<br>- periodontics (clinic)<br>- oral surgery (clinic)<br>- oral examination (clinic)
	**---------- Prosthodontic clinic ----------**						
**6 (1)**	**---------- Prosthodontic clinic ----------**						- restorative (clinic)<br>- endodontics (clinic)<br>- periodontics (clinic)<br>- oral surgery (clinic)<br>- oral examination (clinic)
