# Supplementary material for: Perspective of Undergraduate Dental Students on Horizontal and Vertical Integrations in Prosthodontics Education: A Mixed‐Methods Approach
Source: Int J Dent. 2026 Aug 12;2026:9095160. doi: 10.1155/ijod/9095160 (PMC13469755; doi:10.1155/ijod/9095160)
Supplement: Supplementary file 3 [file IJOD-2026-9095160-s003.docx]

**Supplementary File 3.** Survey Questionnaires

**Part I: Personal information**

Sex:	❒ Male	❒ Female	❒ Not indicated

Current year of education:

❒ 3^rd^ year	❒ 4^th^ year	❒ 5^th^ year	❒ 6^th^ year

❒ recent graduates (academic year 2021 or 2022), not yet pursuing higher education

❒ recent graduates (academic year 2021 or 2022), pursuing higher education

**Part II. Closed-ended questions**

**Questionnaire for clinical students (5th- and 6^th^- year students) and graduates**

Please rate the importance of the following subjects for understanding and providing fixed and removable prosthodontic treatment to patients during the undergraduate prosthodontic course.
*(Please select one answer for each subject)*

| Items | Subjects | Highly essential [3] | Moderately essential [2] | Less essential [1] | Not essential at all [0] |
| --- | --- | --- | --- | --- | --- |
| 1 | Dental anatomy |  |  |  |  |
| 2 | Oral anatomy |  |  |  |  |
| 3 | Human anatomy |  |  |  |  |
| 4 | Histology |  |  |  |  |
| 5 | Biochemistry |  |  |  |  |
| 6 | Physiology |  |  |  |  |
| 7 | Microbiology and immunology |  |  |  |  |
| 8 | Pharmacology |  |  |  |  |
| 9 | General medicine |  |  |  |  |
| 10 | Oral pathology |  |  |  |  |
| 11 | Oral examination |  |  |  |  |
| 12 | Radiology |  |  |  |  |
| 13 | Occlusion |  |  |  |  |
| 14 | Dental materials |  |  |  |  |
| 15 | Operative dentistry |  |  |  |  |
| 16 | Endodontics |  |  |  |  |
| 17 | Community dentistry & Oral epidemiology |  |  |  |  |
| 18 | Oral surgery |  |  |  |  |
| 19 | Restorative dentistry |  |  |  |  |
| 20 | Pediatric dentistry |  |  |  |  |

**Questionnaire for clinical students (4th-year students)
*Note: Fourth-year students have completed all lecture courses in the following subjects but have not yet fully enrolled in clinical courses.***

Please rate the importance of the following subjects for understanding prosthodontic lectures and providing prosthodontic treatment in the undergraduate prosthodontic course.

(Please select one answer for each subject.)

| Items | Subjects | Highly essential [3] | Moderately essential [2] | Less essential [1] | Not essential at all [0] |
| --- | --- | --- | --- | --- | --- |
| 1 | Dental anatomy |  |  |  |  |
| 2 | Oral anatomy |  |  |  |  |
| 3 | Human anatomy |  |  |  |  |
| 4 | Histology |  |  |  |  |
| 5 | Biochemistry |  |  |  |  |
| 6 | Physiology |  |  |  |  |
| 7 | Microbiology and immunology |  |  |  |  |
| 8 | Pharmacology |  |  |  |  |
| 9 | General medicine |  |  |  |  |
| 10 | Oral pathology |  |  |  |  |
| 11 | Oral examination |  |  |  |  |
| 12 | Radiology |  |  |  |  |
| 13 | Occlusion |  |  |  |  |
| 14 | Dental materials |  |  |  |  |
| 15 | Operative dentistry |  |  |  |  |
| 16 | Endodontics |  |  |  |  |
| 17 | Community dentistry & Oral epidemiology |  |  |  |  |
| 18 | Oral surgery |  |  |  |  |
| 19 | Restorative dentistry |  |  |  |  |
| 20 | Pediatric dentistry |  |  |  |  |
