# Supplementary material for: Perspective of Undergraduate Dental Students on Horizontal and Vertical Integrations in Prosthodontics Education: A Mixed‐Methods Approach
Source: Int J Dent. 2026 Aug 12;2026:9095160. doi: 10.1155/ijod/9095160 (PMC13469755; doi:10.1155/ijod/9095160)
Supplement: Supplementary file 4 [file IJOD-2026-9095160-s004.docx]

**Supplementary File 4.** Focus Group Interview Questions

**Part 1	Questionnaire development**

**Research Hypothesis**

Regarding outcome-based education, curriculum content is outlined based on desirable program outcomes which are formulated using stakeholder’s needs and contextual analysis (Supplementary File 1). Then, courses are designed and arranged into a curriculum structure (Supplementary File 2). Connections amongst courses are illustrated as vertical and horizontal integration demonstrating that, within the constructed curriculum sequences, students are ensured to gradually develop professional competencies. Focusing on prosthodontic competency, identifying and understanding the connections between foundational courses, prosthodontic courses, and courses from other disciplines can help design effective educational strategies to support students develop prosthodontic competency.

**Theoretical foundation:**

1. Literature reviews – including the nature of prosthodontics, relationship between prosthodontics and other disciplines, vertical and horizontal integration, threshold concepts (barriers to cognitive development), tacit knowledge (barriers to psychomotor development).
2. Results from the quantitative questionnaires – essentiality of foundational courses and courses from other disciplines toward learning prosthodontics courses.

**Type: Semi-structured interviews**

1. Opening questions – for ice breaking and reflection-on-action (review what students have learnt and practices).
2. Key questions – for identifying students’ experience and perception in

(1) learning prosthodontic courses, and

(2) how understanding foundational courses and courses from other disciplines support learning prosthodontics courses and developing prosthodontic competency.

3. Probing questions – for exploring in-depth information during the discussion
4. Closing questions – for adding further discussion (if necessary)

**Part 2	Focus group interview questions**

| **Opening questions** | |
| --- | --- |
| Ice-breaking | - Are you happy with your current study? Why? |
|  | - What subjects are you studying in this academic year? |
| Reflection-on-action | - Till now, what have you studied or practiced in relation to prosthodontics? |
|  | - How do you feel about your prosthodontic knowledge and performance (in either lab or clinic)? |
| **Key questions** | |
| Prosthodontic learning and competency development | - Please describe your experiences in learning/practicing in prosthodontic courses.   Probing questions (examples)   - What have you done well? Why? - What knowledge/practice do you think you need improvement? Why? |
| Connections between prosthodontic courses and other courses | - From your experience, what are connections between prosthodontic courses and other courses in the program?   Probing questions (examples)   - What foundation knowledge/practices are required for effective prosthodontic learning? Please explain. - What knowledge/practices in other disciplines are required for effective prosthodontic learning? Please explain. |
| Difficulties in learning prosthodontics | - What prosthodontic contents/topics are difficult to learn or apply in practice? Why?   Probing questions (examples)   - What are the most difficult parts to learn? Please explain. - What are the parts that require understanding of topics from other subjects/courses? Please explain. - What are the parts that you have never forgotten since you studied/learnt? Please explain. |
| Strategies for overcoming barriers for prosthodontic learning | - How did you learn difficult prosthodontic contents/topics? What techniques did you use?   Probing questions (examples)   - Did you overcome the difficulties in learning/by yourself or by the help of others? How? |
| Curriculum improvement | - From your experience, what are the most appropriate ways to help you learn prosthodontic contents/topics effectively? Please explain.   Probing questions (examples)   - How can educators help or support you in this aspect? - How can the prosthodontic courses or other courses be revised to ensure that you can learn prosthodontics effectively? |
| **Closing questions** | |
| Final thought | - Do you have anything to add to our discussion today? - Do you have any questions to ask the researchers? |
